# Evaluation of nikkomycin Z with frequent oral administration in an experimental model of central nervous system coccidioidomycosis

**DOI:** 10.1128/spectrum.01356-24

**Published:** 2024-08-20

**Authors:** Nathan P. Wiederhold, Laura K. Najvar, Rosie Jaramillo, Marcos Olivo, David J. Larwood, Thomas F. Patterson

**Affiliations:** 1 Department of Pathology and Laboratory Medicine, Fungus Testing Laboratory, University of Texas Health Science Center at San Antonio, San Antonio, Texas, USA; 2 Department of Medicine, Division of Infectious Diseases, University of Texas Health Science Center at San Antonio, San Antonio, Texas, USA; 3 Valley Fever Solutions, Inc., Tucson, Arizona, USA; Broad Institute, Cambridge, Massachusetts, USA

**Keywords:** nikkomycin Z, *Coccidioides *species, coccidioidomycosis

## Abstract

We evaluated the *in vivo* activity of nikkomycin Z against central nervous system coccidioidomycosis. Mice were inoculated intracranially with arthroconidia of *Coccidoides immitis*, and treatment with nikkomycin Z (50, 100, or 300 mg/kg orally TID) or fluconazole (25 mg/kg orally BID) began 2 days later. Each dose of nikkomycin Z and fluconazole significantly improved survival and reduced brain fungal burden compared with vehicle control. Further studies of nikkomycin Z against coccidioidomycosis are warranted.

**IMPORTANCE:**

*Coccidioides* species are endemic fungi that are capable of causing disease in patients with various comorbidities, as well as in otherwise healthy individuals. Treatment options for coccidioidomycosis are suboptimal, as azole antifungals may be limited by drug interactions and adverse effects due to interactions with enzymes found in humans and other mammals. Nikkomycin Z is an investigational agent that works against a target specific to the fungal cell wall (chitin), which is not present in the cells of humans or other mammals. In this study, we show that frequent oral administration of nikkomycin Z is effective in an experimental model of central nervous system coccidioidomycosis. Further studies of nikkomycin Z against coccidioidomycosis may be warranted.

## OBSERVATION

Coccidioidomycosis is an invasive mycosis that is caused by the dimorphic fungi *Cocciddioides immitis* and *C. posadasii*. These fungi are endemic to warm, arid regions in the Western hemisphere. In the U.S., this includes areas of Arizona, California, New Mexico, Texas, Utah, and Washington State (1, 2), although this endemic area appears to be increasing (3, 4). *Coccidioides* arthroconidia are inhaled into the lungs resulting in asymptomatic-to-mild respiratory infections in most individuals. However, disseminated disease, including central nervous system infections, can occur in between 1% and 3% of individuals (5). Fluconazole is often used in individuals with coccidioidal meningitis due to its safety profile and good CNS penetration (5). However, therapy must be continued indefinitely, as relapses occur when this azole is stopped (6), and there is concern for reduced *in vitro* susceptibility or resistance to fluconazole in *Coccidioides* clinical isolates (7).

Nikkomycin Z is an investigational agent that acts as a competitive inhibitor of chitin synthase, thereby reducing the levels of chitin, an essential polymer of the cell wall of fungi that is not found in humans (8, 9). *In vitro* nikkomycin Z has been shown to disrupt the cell wall and internal structure of the spherule–endospore phase of *C. immitis* with cell lysis also being observed when assessed by electron microscopy (9). We evaluated the *in vivo* activity of nikkomycin Z against CNS coccidioidomycosis caused by *C. immitis*.

Immunocompetent male Institute for Cancer Research (ICR) mice (10 per group; Envigo, Indianapolis, IN) were anesthetized with isoflurane and underwent intracranial inoculation with arthroconidia of a clinical *C. immitis* strain (UTHSCSA DI17-143; 100 to 4,400 arthroconidia/mouse) as previously described (10, 11). *In vitro* testing was performed against a limited number of *C. immitis* strains (*n* = 3) by broth macrodilution (12). Nikkomycin Z MICs ranged from 0.125 to 4 µg/mL using the 50% inhibition of growth endpoint and from 0.5 to >16 µg/mL using the 100% inhibition of growth endpoint and were highest against the strain used to establish infection in the *in vivo* model. *In vitro* testing was repeated against this isolate, and the results were consistent between experiments. Both fungal burden and survival arms were included. Forty-eight hours after inoculation, treatment began and continued for 7 and 14 days in the fungal burden arm and survival arm, respectively. Dosing was performed by oral gavage, and groups consisted of vehicle control (sterile water), nikkomycin Z at doses of 50, 100, and 300 mg/kg three times daily, or fluconazole 25 mg/kg twice daily. Animal studies were performed according to Public Health Service (PHS) animal welfare guidelines under an approved institutional animal care and use protocol and at BSL3. In the survival arm, once therapy had stopped, mice were monitored off therapy until day 30 post-inoculation. Animals that appeared moribund by pre-specified criteria were humanely euthanized, and death was recorded as occurring the next day. Brain fungal burden was measured in the fungal burden arm on day 9 post-inoculation (1 day after the last treatment dose) and in the survival arm at the time of euthanasia or the predefined study endpoint (day 30). Brains were collected, weighed, and homogenized in sterile saline. The homogenates were diluted, and aliquots were plated onto potato dextrose agar. The number of CFUs was counted after 72–96 h of incubation at 37°C, and fungal burden (CFU/g tissue) was determined. Survival was plotted by Kaplan–Meier analysis, and differences in median and percent survival to day 30 were analyzed using log-rank and Fisher’s exact test, respectively. Differences in brain fungal burden were assessed using the Kruskal–Wallis test with Dunn’s correction for multiple comparisons. P-values of ≤0.05 were considered statistically significant.

**Fig 1:**
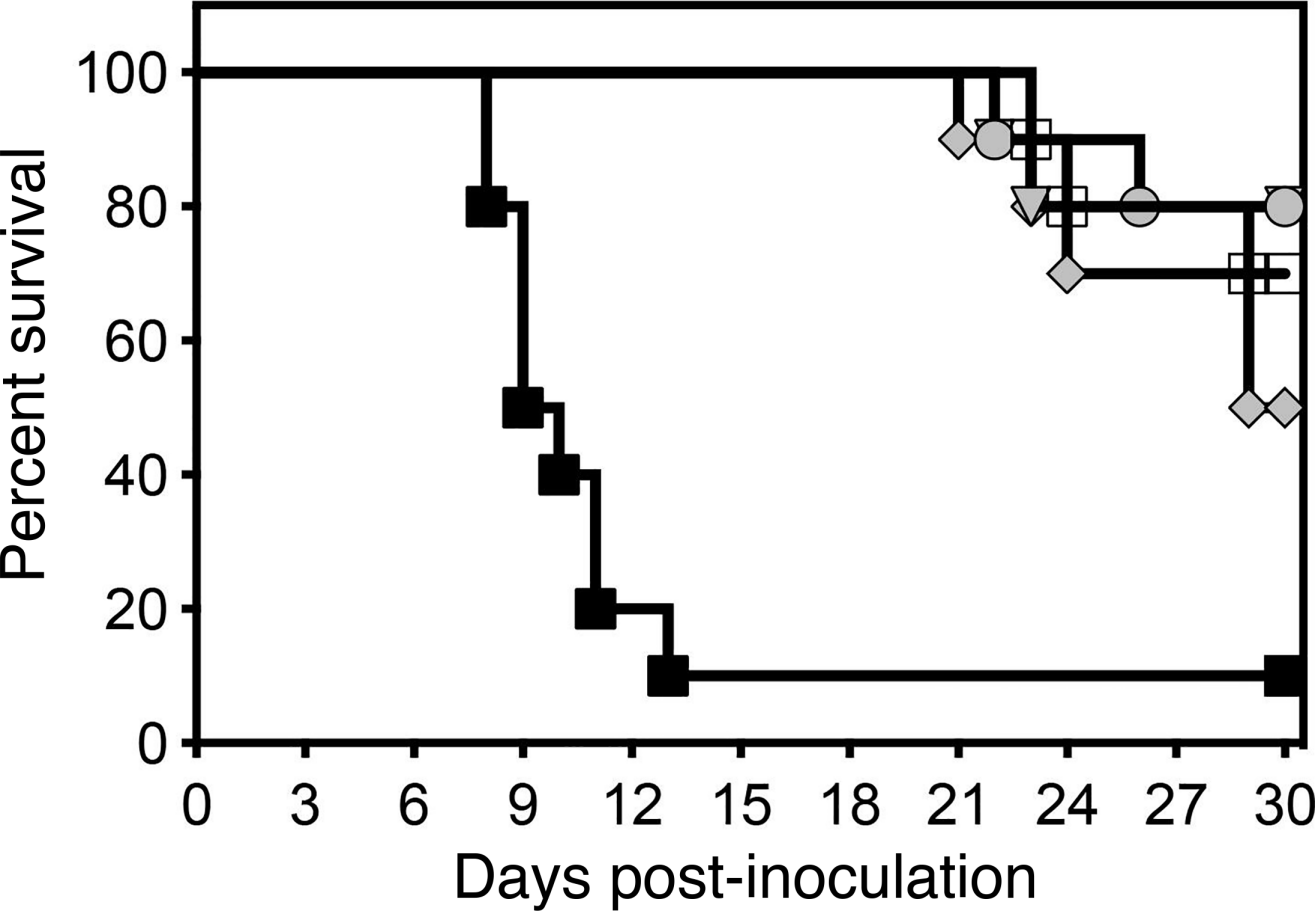
Survival curves in mice with CNS coccidioidomycosis and treated with nikkomycin Z or fluconazole. Treatment was started 2 days post-inoculation and was continued for 14 days. Mice were then monitored off therapy until day 30. Black square – vehicle control (sterile water); white square – nikkomycin Z 50 mg/kg PO TID; gray circle – nikkomycin Z 100 mg/kg PO TID; inverted gray triangle – nikkomycin Z 300 mg/kg PO TID; gray diamond – fluconazole 25 mg/kg PO BID.

**Fig 2:**
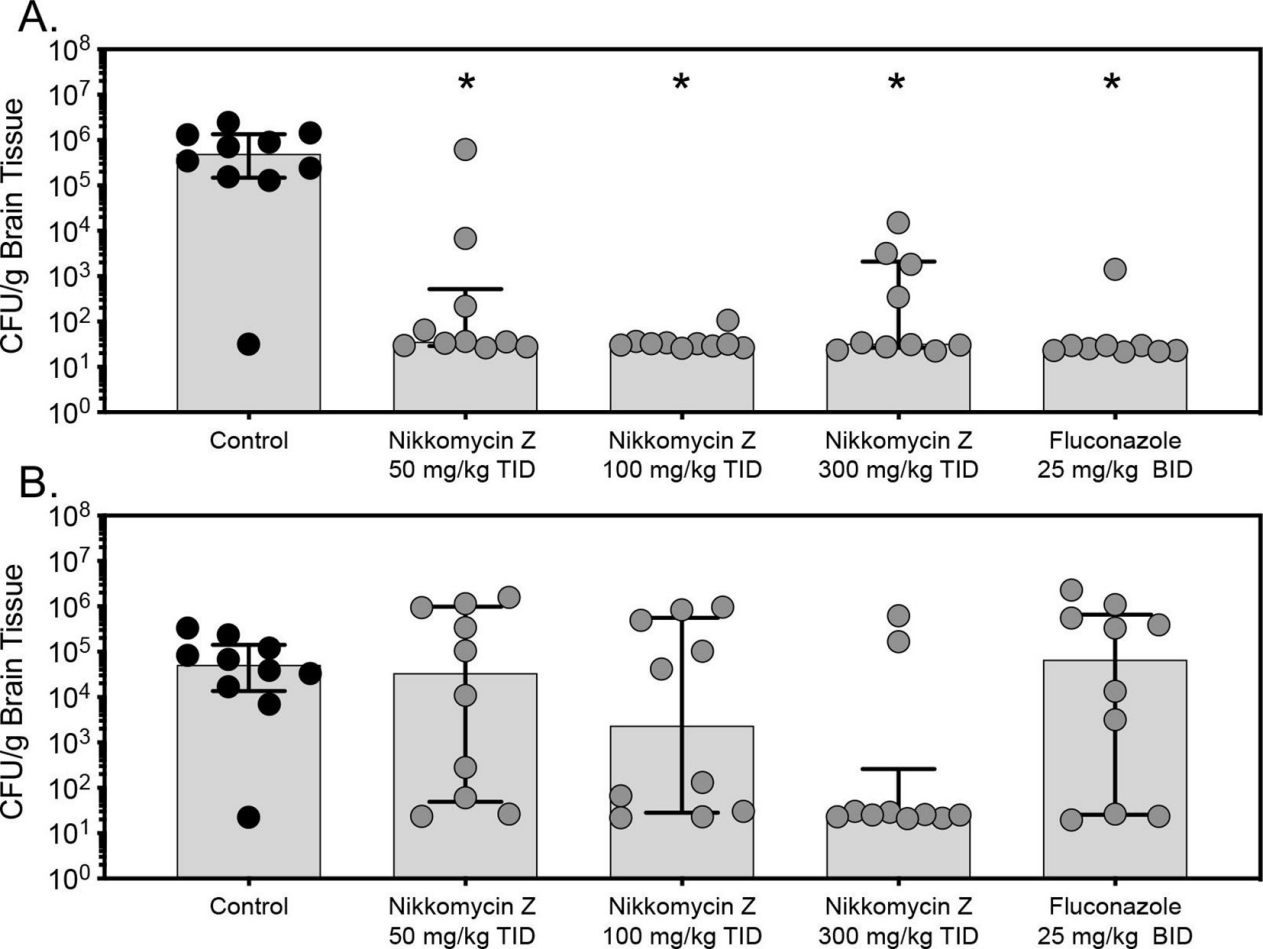
Brain tissue fungal burden (CFU/g) in mice with CNS coccidioidomycosis secondary to *C. immitis* in the fungal burden (**A**) and survival (**B**) arms. In the fungal burden arm, treatment continued for 7 days, and brains were collected 1 day after treatment stopped. In the survival arm, treatment continued for 14 days, and colony-forming units were measured at the time of morbidity or at the pre-specified study endpoint (day 30). Median values, interquartile ranges, and individual data points are shown. *P < 0.05 vs. Control.

Nikkomycin Z was effective at improving survival in this experimental model of CNS coccidioidomycosis. At each dose level of this investigational agent, both median survival (>30 days) and percent survival (range 70%–80%) at the study endpoint, day 30 post-inoculation, were significantly improved compared with the vehicle control group (9.5 days and 10%; *P* ≤ 0.02 for all comparisons) (Fig. 1). Similar results were also observed with fluconazole (29.5 days and 50%). In the fungal burden arm, treatment with nikkomycin Z also significantly reduced brain fungal burden on day 9 post-inoculation (median range, 1.51–1.55 log_10_ CFU/g) compared with vehicle control (5.66 log_10_ CFU/g; *P* ≤ 0.05 for all comparisons) (Fig. 2A). Similar results were also observed with fluconazole (1.40 log_10_ CFU/g; *P* < 0.001). In the survival arm, rebounds in fungal burden were observed in the nikkomycin Z 50 mg/kg and 100 mg/kg groups (median, 4.53 and 3.37 log_10_ CFU/g) and fluconazole group (4.82 log_10_ CFU/g), and this was primarily driven by higher fungal burden in mice that succumbed to infection before day 30. In contrast, brain fungal burden remained numerically low in the nikkomycin Z 300 mg/kg group (1.41 log_10_ CFU/g) compared with the vehicle control group (4.71 log_10_ CFU/g), although this difference was not statistically significant.

These results are consistent with those reported by others in murine models of pulmonary, disseminated, and CNS coccidioidomycosis. In these studies, infection was established using *C. posadasii* Silveira strain (ATCC 28868), and nikkomycin Z was administered at various doses ranging from 20 to 6,000 mg/kg/day via drinking water or oral gavage (9, 13–15). When administered via drinking water, reductions in fungal burden in various organs were reported, although the exact amounts each animal received could not be determined due to this route of administration. In the current study, nikkomycin Z was given by oral gavage to administer a defined dose, and this was done at a frequency of three times daily due to the rapid clearance of this investigational agent in mice and humans, with a half-life in humans between 2.1 and 2.5 h following oral administration (9, 16). With this route and frequency of dosing, we observed improvements in survival and reductions in fungal burden. These results are particularly encouraging given the elevated MIC of nikkomycin Z against the isolate used to establish infection in this model. Nikkomycin Z also has *in vivo* activity against other dimorphic fungi, including *Blastomyces dermatitidis* and *Histoplasma capsulatum* (9, 17). Similar to the results of the current study, improvements in survival and reductions in fungal burden were observed in mice infected with these fungi and treated with nikkomycin Z. Overall, these results suggest that further studies of nikkomycin Z against coccidioidomycosis may be warranted.
